# Cooperative interaction of CTGF and TGF-β in animal models of fibrotic disease

**DOI:** 10.1186/1755-1536-4-4

**Published:** 2011-02-01

**Authors:** Qingjian Wang, William Usinger, Blake Nichols, Julia Gray, Leon Xu, Todd W Seeley, Mitch Brenner, Guangjie Guo, Weihua Zhang, Noelynn Oliver, Al Lin, David Yeowell

**Affiliations:** 1 FibroGen Inc., 409 Illinois St., San Francisco, CA 94158, USA

## Abstract

**Background:**

Connective tissue growth factor (CTGF) is widely thought to promote the development of fibrosis in collaboration with transforming growth factor (TGF)-β; however, most of the evidence for its involvement comes from correlative and culture-based studies. In this study, the importance of CTGF in tissue fibrosis was directly examined in three murine models of fibrotic disease: a novel model of multiorgan fibrosis induced by repeated intraperitoneal injections of CTGF and TGF-β2; the unilateral ureteral obstruction (UUO) renal fibrosis model; and an intratracheal bleomycin instillation model of pulmonary fibrosis.

**Results:**

Intraperitoneal coadministration of CTGF and TGF-β2 elicited a profound fibrotic response that was inhibited by the human anti-CTGF antibody FG-3019, as indicated by the ability of FG-3019 to ameliorate the histologic signs of fibrosis and reduce the otherwise increased hydroxyproline:proline (Hyp:Pro) ratios by 25% in kidney (*P *< 0.05), 30% in liver (*P *< 0.01) and 63% in lung (*P *< 0.05). Moreover, administration of either cytokine alone failed to elicit a fibrotic response, thus demonstrating that CTGF is both necessary and sufficient to initiate fibrosis in the presence of TGF-β and *vice versa*. In keeping with this requirement for CTGF function in fibrosis, FG-3019 also reduced the renal Hyp:Pro response up to 20% after UUO (*P *< 0.05). In bleomycin-injured animals, a similar trend towards a FG-3019 treatment effect was observed (38% reduction in total lung Hyp, *P *= 0.056). Thus, FG-3019 antibody treatment consistently reduced excessive collagen deposition and the pathologic severity of fibrosis in all models.

**Conclusion:**

Cooperative interactions between CTGF and TGF-β signaling are required to elicit overt tissue fibrosis. This interdependence and the observed anti-fibrotic effects of FG-3019 indicate that anti-CTGF therapy may provide therapeutic benefit in different forms of fibroproliferative disease.

## Background

Fibroproliferative diseases, including chronic pulmonary, hepatic, renal and vascular fibrosis, contribute to nearly half of all deaths in the USA[1,2]. These disorders frequently affect multiple organ systems, complicating the elucidation of their underlying pathogenesis and hindering the development of effective treatments. Nevertheless, two secreted factors, transforming growth factor (TGF-β and connective tissue growth factor (CTGF), are widely regarded as universal mediators of fibrogenesis, although the precise mechanisms that underlie their concerted effects remain unclear [3-8]. Because TGF-β is a potent inducer of CTGF, most models postulate that CTGF acts as a downstream mediator of TGF-β activity, whereas other studies support an interdependent rather than merely sequential relationship between TGF-β and CTGF and their respective profibrotic activities [9-11].

CTGF is a multifunctional heparin-binding glycoprotein that is normally expressed at low levels but dramatically enriched in virtually all fibrotic conditions [8]. Cell-based studies have shown that CTGF regulates multiple processes that contribute to fibrogenesis, including cell proliferation, migration, adhesion, survival and extracellular matrix production; that it does so as both a downstream and cooperative mediator of TGF-β signaling; and that it affects a variety of cell types that participate in the fibrogenic process, regardless of tissue origin, including mesenchymal stem cells, hepatic stellate cells, renal podocytes, mesangial cells, parietal and tubular epithelial cells, pulmonary type II alveolar cells, mesothelial cells, vascular smooth muscle cells, endothelial cells, cardiomyocytes, pericytes and fibroblasts [4]. Moreover, correlative studies in diseased human tissues indicate important links between CTGF and TGF-β in a number of fibrotic disease states, including diabetic nephropathy, idiopathic and non-idiopathic pulmonary fibrosis, liver fibrosis, skin fibrosis (including keloids and systemic sclerosis), atherosclerosis, congestive heart failure, pancreatitis and various forms of malignant disease [3,8].

Data from animal models of human disease also support the importance of CTGF and its interactions with TGF-β in fibrosis. The small interfering (si)RNA-mediated knockdown of CTGF, for example, has been shown to suppress fibrotic responses in three models of liver fibrosis [5,12,13] and in a chronic allograft nephropathy model [14]. Similarly, antisense-mediated knockdown of CTGF has been shown to suppress fibrosis or its indicators in models of type 1 and type 2 diabetic nephropathy [15], unilateral ureteral obstruction (UUO) and subtotal (5/6) nephrectomy-induced kidney fibrosis [16,17], chemically induced liver fibrosis [18], hypertrophic scarring after dermal wound repair [19], and capsular scarring and contracture surrounding breast implants [20]. Notably, TGF-β induction was also reduced after siRNA- and antisense-mediated CTGF knockdown in five of six models in which it was examined [5,12,13,15,16,18], thus indicating that CTGF activity directly or indirectly enhances the expression of its own inducer in a positive feedback manner. In addition, the expression of known TGF-β targets was inhibited by antisense CTGF knockdown in the 5/6 nephrectomy model despite concurrent overexpression of a TGF-β transgene, suggesting that CTGF also promotes TGF-β activity in a fibrotic setting [17]. Thus, CTGF may influence both the level and function of TGF-β and like CTGF, the targeted inhibition of TGF-β itself attenuates fibrogenesis in these and other disease models. However, the myriad crucial functions of TFG-β make it unattractive as a direct target for antifibrotic therapy [3].

In the work described here, a novel CTGF and TGF-β synergy model was developed based on the prior observation that simultaneous or serial subcutaneous coadministration of TGF-β3 and CTGF elicited the formation of persistent fibrotic lesions in neonatal mice, whereas administration of either cytokine alone failed to produce any persistent effect [21]. In this study, we extended these findings to a model in which intraperitoneal co-injections of TGF-β2 and recombinant human CTGF produced profound peritoneal fibrosis, abdominal adhesions, and disseminated fibrosis in vital organs, including lung, heart, kidney and liver. As had been shown in the earlier subcutaneous fibrosis model, fibrosis in the intraperitoneal administration model also required the coadministration of both TGF-β and CTGF, thus demonstrating a robust cooperative interaction between these two factors in the genesis and maintenance of a fibrotic response. Furthermore, FG-3019, a fully human recombinant monoclonal antibody against human CTGF, blocked fibrosis in this model and in two additional clinically relevant rodent fibrosis models, the UUO model of kidney fibrosis and the bleomycin model of lung fibrosis, both of which also respond to TGF-β/Smad-targeted interventions [22-24]. Thus, treatment with a CTGF-blocking antibody reduced the severity of fibrosis in all three models, thereby indicating that anti-CTGF therapy may be clinically beneficial in diverse fibroproliferative diseases.

## Results

### Production and characterization of FG-3019 monoclonal antibody

**Figure 1:**
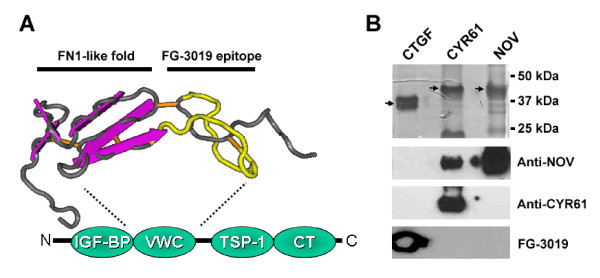
**The FG-3019 binding epitope overlaps with the VWC domain of connective tissue growth factor (CTGF)**. **(A) **The region of the FG-3019 binding epitope (CTGF amino acids 142 to 157, indicated in yellow) is superimposed on the structure of the collagen IIa VWC domain [50]. By sequence homology, the general structural features of this domain are predicted to be conserved in CTGF and other CCN (connective tissue growth factor) proteins [51]. The predicted FG-3019 binding site lies outside of a fibronectin-1-like module within the VWC domain of CTGF. A physical interaction between the VWC domain of *Xenopus *CTGF and TGF-β family members TGF-β1 and bone morphogenetic protein (BMP)-4 was reported [9]. The location of the VWC domain relative to the insulin-like growth factor binding protein (IGF-BP), thrombospondin-1 (TSP1) and C-terminal cysteine knot (CT) homology domains of CCN family members is also indicated. **(B) **Recombinant human CTGF, CYR61 and NOV proteins (visualized by Coomassie blue in the upper panel) were analyzed by western blot using FG-3019, anti-CYR61 and anti-NOV antibodies. FG-3019 specifically bound CTGF without crossreacting with CYR61 or NOV.

FG-3019, a fully human recombinant DNA-derived CTGF-reactive monoclonal IgG1/κ antibody, was isolated and cloned from genetically engineered mice expressing human immunoglobulin transgenes (Medarex, Princeton, NJ, USA) [25], which had been immunized with recombinant human CTGF. Using engineered CTGF fusion proteins and CTGF proteolysis fragments, FG-3019 binding activity was mapped to the cysteine-rich von Willebrand Factor type C (VWC) domain of CTGF (Figure 1A). Synthetic peptides derived from the VWC domain sequences of CTGF efficiently displaced FG-3019 in competitive CTGF ELISAs, including a peptide corresponding to CTGF amino acids 135-157, suggesting that these sequences participate in the recognition and binding of CTGF by FG-3019. This segment of the CTGF primary sequence is poorly conserved between related CCN family proteins, including NOV and CYR61 but well conserved within CTGF proteins encoded by diverse mammalian species [26]. Indeed, FG-3019 binds CTGF from a variety of mammalian species, including rodents, and radioimmunoassays indicate that it binds human and rat CTGF with *K*_d _values of approximately 0.1 and 0.3 nmol/l, respectively (data not shown). To confirm the specificity of FG-3019 towards CTGF, as opposed to the structurally related CCN proteins CYR61 or NOV, binding of FG-3019 to recombinant proteins was compared by immunoblot analysis; FG-3019 was indeed avidly associated with CTGF, but not with CYR61 or NOV (Figure 1B). This result was also confirmed by ELISA (data not shown).

### Profibrotic effects of TGFβ2 and CTGF coadministration in neonatal mice

**Figure 2:**
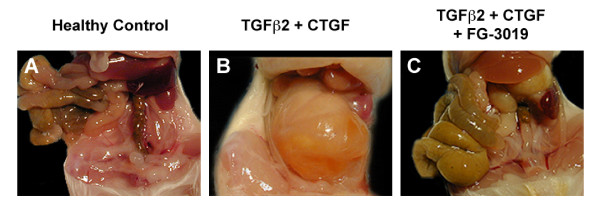
**Gross anatomy in the transforming growth factor-β/connective tissue growth factor (TGF-β/CTGF) synergy model**. Peritoneal cavities of **(A) **healthy control animals, **(B) **animals coadministered CTGF and TGF-β2, and **(C) **animals coadministered CTGF and TGF-β2 and treated with anti-CTGF antibody FG-3019. Extensive fibrotic membranes surrounded most organs in the abdominal cavity in vehicle-treated mice that received both TGF-β2 and CTGF. A substantial reduction in gross fibrotic pathology was observed in mice that received TGF-β2 and CTGF plus FG-3019.

A novel model of multiorgan fibrosis was developed, involving the repeated intraperitoneal injection of newborn mice with a combination of TGF-β2 and CTGF. Whereas the gross anatomy of control mice treated with vehicle, TGF-β2 or CTGF alone appeared normal, animals treated with both cytokines together exhibited a profound thickening of the abdominal wall and peritoneal membrane (Figure 2A, B). In severe cases, a thickened fibrous membrane covered the surface of almost all the abdominal organs, and fibrous adhesions were readily apparent between adjacent organs or between otherwise free tissues and the abdominal wall. Organ fibrosis was quantitatively scored by comparing the Hyp:Pro ratios within tissues, which is a standard measure of collagen content relative to total organ protein. Hyp:Pro ratios in the kidney, liver, lung and heart were substantially increased after coadministration of TGF-β2 and CTGF, whereas treatment with either cytokine alone did not increase Hyp:Pro ratios relative to vehicle controls (Table 1), thus demonstrating a clear interaction between CTGF and TGF-β in this model. By these measures, Hyp:Pro changes after coadministration of TGF-β2 and CTGF were most dramatic in kidney and liver (increases of 65% and 160%, respectively, over control,), with more moderate but significant increases of 34% and 11% being seen in heart and lung, respectively (*P *< 0.001 in kidney, liver and heart, *P *< 0.01 in lung). Because the latter tissues lie outside the peritoneal cavity, this significant increase in their relative Hyp:Pro content indicates that the intraperitoneal coadministration of TGF-β2 and CTGF elicited a systemic rather than purely local fibrotic response. The coadministration of CTGF and TGF-β2 was also associated with a 28% reduction in final body weight (mean ± SE 7.62 ± 0.10 g) compared with healthy controls (10.59 ± 0.24 g), which was significant (*P *< 0.001). In addition, the severe intra-abdominal fibrosis in these animals appeared to be associated with compromised bowel motility, as indicated by increased accumulation of fecal material in their intestines (data not shown).

**Table 1:** Hydroxyproline:proline ratio by treatment group and organ in the TGF-β/CTGF synergy model

Organ	Control	Treatments			
	-	TGF-β2	-	TGF-β2	TGF-β2

	-	-	CTGF	CTGF	CTGF

	-	-	-	-	FG-3019

Kidney	0.057 ± 0.001	0.061 ± 0.002	0.059 ± 0.001	0.094 ± 0.0051	0.085 ± 0.003^1,3^

Liver	0.015 ± 0.001	0.017 ± 0.001	0.016 ± 0.002	0.039 ± 0.003^1^	0.032 ± 0.002^1,4^

Lung	0.079 ± 0.002	0.078 ± 0.002	0.083 ± 0.001	0.088 ± 0.001^2^	0.083 ± 0.002^,3^

Heart	0.041 ± 0.001	0.044 ± 0.002	0.046 ± 0.002	0.055 ± 0.002^1^	0.053 ± 0.003^1^

Data shown represent mean ± SEM, *n *= 6 to 10.

^1^*P *< 0.001, ^2^*P *< 0.01 compared with healthy control group, one way analysis of variance, Fisher's least significant difference.

^3^*P *< 0.05, ^4^*P *< 0.01 compared with transforming growth factor (TGF)-β2 and connective tissue growth factor (CTGF) coadministration group.

In animals that received both TGF-β2 and CTGF, simultaneous treatment with FG-3019 resulted in substantial visible suppression of the peritoneal membrane and abdominal cavity fibrotic responses (Figure 2C). Overt intra-abdominal adhesions were not noted in these animals. This qualitative reduction in gross fibrotic effect was accompanied by a 22% improvement in the mean final (day 21) body weight in the FG-3019-treated cohort (9.32 ± 0.32 g) compared with animals that received TGF-β2 and CTGF without FG-3019 treatment (*P *< 0.001), although the FG-3019 treated animals were still 12% lighter than healthy controls (*P *< 0.001). FG-3019 treatment also substantially reduced Hyp:Pro ratios in all organs examined. Specifically, the increase in relative Hyp:Pro content (that is, the fibrotic response) elicited by coadministration of TGF-β and CTGF was reduced by 63% in lung (*P *< 0.05), 25% in kidney (*P *< 0.05), 30% in liver (*P *< 0.01) and 15% in heart after FG-3019 treatment (Table 1).

**Figure 3:**
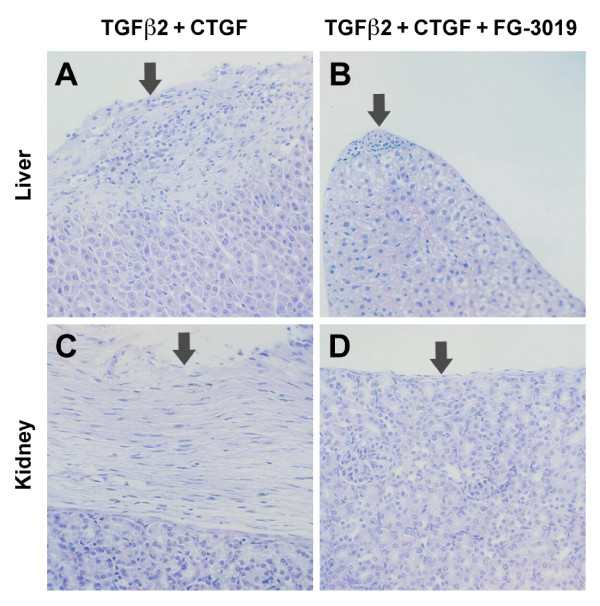
**Hepatic and renal fibrosis in mice receiving transforming growth factor (TGF)-β and connective tissue growth factor (CTGF) is ameliorated by FG-3019 treatment**. **(A, B) **Liver and **(C, D) **kidney of 21-day-old animals coadministered TGF-β2 and CTGF; animals were treated with either **(A, C) **vehicle or **(B, D) **FG-3019. All agents were administered daily for 20 days beginning one day after birth. Arrows indicate regions of robust capsular fibrosis inhibited by FG-3019 treatment. Representative images are shown (all haematoxylin and eosin, original magnification × 200).

The observation that frank fibrosis only occurred after coadministration of both TGF-β and CTGF, as opposed to either cytokine alone, and that it was attenuated by FG-3019 antibody treatment, was also confirmed by an independent histologic analysis (Comparative Biosciences, Inc., Sunnyvale, CA, USA). Microscopic analysis of stained liver sections revealed multifocal fibrosis of the hepatic capsule in eight of eight animals that received both cytokines (Figure 3A, Table 2). In addition, multiple foci of trace or mild parenchymal fibrosis, primarily in periportal liver regions, were present in three animals that received both cytokines. Thus, the response to TGF-β/CTGF coadministration was primarily one of capsular fibrosis with occasional parenchymal involvement. By contrast, histologic evidence of capsular or parenchymal liver fibrosis was entirely absent in all animals that received either CTGF or TGF-β alone, and focal evidence of trace capsular fibrosis without parenchymal fibrosis was seen in only two of eight animals that received FG-3019 (Figure 3B). Thus, the incidence of any degree of fibrosis after dual-cytokine treatment was significantly reduced by CTGF-targeted FG-3019 treatment (Fisher's exact test, *P *= 0.007) and analysis of variance by ranks showed that the histologic liver fibrosis severity scores (0 = no fibrosis, 1 = minimal, 2 = minimal to mild, 3 = mild and 4 = moderate fibrosis) for animals that received both TGF-β2 and CTGF (median 1.5, range 1 to 4) were significantly higher than for animals that received vehicle, TGF-β2 or CTGF alone (all scores = 0; Dunn's test, *P *< 0.001) and that they were significantly reduced by FG-3019 treatment (median 0, range 0 to 1, *P *< 0.01). Likewise, mild to moderate renal capsular fibrosis was observed in three of eight animals that received both cytokines without FG-3019 treatment (Figure 3C), whereas no evidence of fibrosis was seen in any other animal from any other group, including the FG-3019 treatment group (Figure 3D). Fibrosis of the renal parenchyma was not seen in any animal in this study, nor was inflammation noted in any tissue.

**Table 2:** Incidence of histologic liver fibrosis by severity score

Treatment	Degree of liver fibrosis (score)				
	
	None (0)	Minimal (1)	Minimal to mild (2)	Mild (3)	Moderate (4)
Vehicle	9	0	0	0	0

TGF-β2	8	0	0	0	0

CTGF	6	0	0	0	0

TGF-β2 + CTGF	0	4	2	1	1

TGF-β2 + CTGF + FG-3019	6	2	0	0	0

Values indicate the number of mice with each categorical liver fibrosis score.

### Effect of CTGF inhibition in a UUO-induced kidney fibrosis model

Previous studies have demonstrated a direct correlation between CTGF levels and renal pathology in the UUO-induced kidney fibrosis model [16,27]. In the current study, ligated kidneys of vehicle-treated animals were compared with non-ligated control kidneys of the same animals to determine the fibrotic effect of UUO using the standard tissue Hyp:Pro ratio to assess the fibrotic response. In addition, to determine whether disruption of CTGF signaling inhibits the UUO-induced fibrotic response, ligated kidneys of animals treated for 2 weeks with either vehicle or FG-3019 were compared.

**Figure 4:**
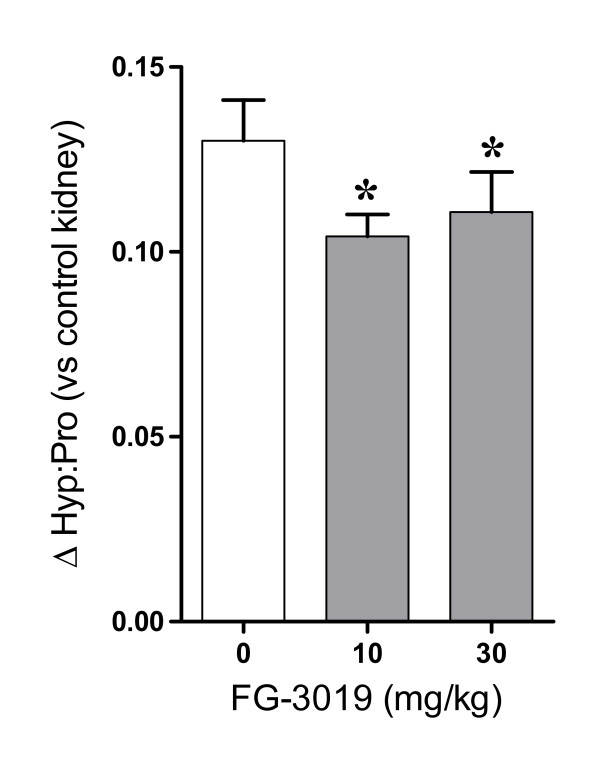
**FG-3019 inhibits renal collagen deposition after unilateral ureteral obstruction (UUO)**. Hydroxyproline:proline (Hyp:Pro) ratios in renal tissues from UUO mice are expressed as the mean change above the mean Hyp:Pro ratio of unligated control kidneys (0.0446 ± 0.0005, *n *= 35). Mean renal Hyp:Pro ratio increases for the 10 and 30 mg/kg FG-3019 treatment groups (gray bars) were 20% and 15% lower than for the UUO kidneys of vehicle-treated controls (white bar), respectively. *P < 0.05 compared with UUO + vehicle (analysis of variance, Fisher's least significant difference).

In vehicle-treated control animals, UUO induced a clear fibrotic effect, as indicated by a fourfold increase in the mean Hyp:Pro ratio in ligated kidneys compared with non-ligated control kidneys (0.175 ± 0.011 vs. 0.045 ± 0.0005; *P *< 0.001). The kidneys of animals treated with 10 and 30 mg/kg FG-3019 were significantly protected from UUO-induced fibrosis, as indicated by reduction of 20% and 15%, respectively, in their induced Hyp:Pro ratios compared with untreated UUO control mice (*P *< 0.05) (Figure 4), respectively. Histologic analysis using trichrome staining revealed interstitial fibrosis, fibroblast proliferation and increased collagen accumulation in all obstructed kidneys regardless of treatment, with a slight but subjective decrease in the amount of fibrosis seen in the 10 mg/kg FG-3019 treatment group (not shown). Increased trichrome staining was associated with the fibroblast proliferation, and was noted in the interstitium of the cortex, the surrounding glomeruli and the medullary regions of all obstructed kidneys. Marked hydronephrosis, characterized by tubular dilation and tubular epithelial loss, was also seen throughout the cortex in all obstructed kidneys regardless of treatment. In addition, multifocal lymphocytic infiltrates were commonly observed in the interstitium of obstructed kidneys, particularly in perivascular regions; however, this was also unaffected by treatment.

### Effect of FG-3019 in a bleomycin-induced pulmonary fibrosis model

To confirm that CTGF actively contributes to the pathogenesis of pulmonary fibrosis, and that CTGF inhibition may alleviate the pulmonary fibrotic response, the effect of FG-3019 treatment was examined in a bleomycin-induced lung fibrosis model [24,28]. Bleomycin-treated animals were compared with healthy controls to determine the fibrotic effect of pulmonary bleomycin instillation. Bleomycin-treated animals treated for 2 weeks with either vehicle or FG-3019 were further compared, to investigate the importance of CTGF signaling in pulmonary fibrogenesis. Fibrosis in this model was scored by the standard measure of total lung Hyp, which takes into account the variable Pro content in lung, which is often unrelated to changes in total lung collagen content.

**Figure 5:**
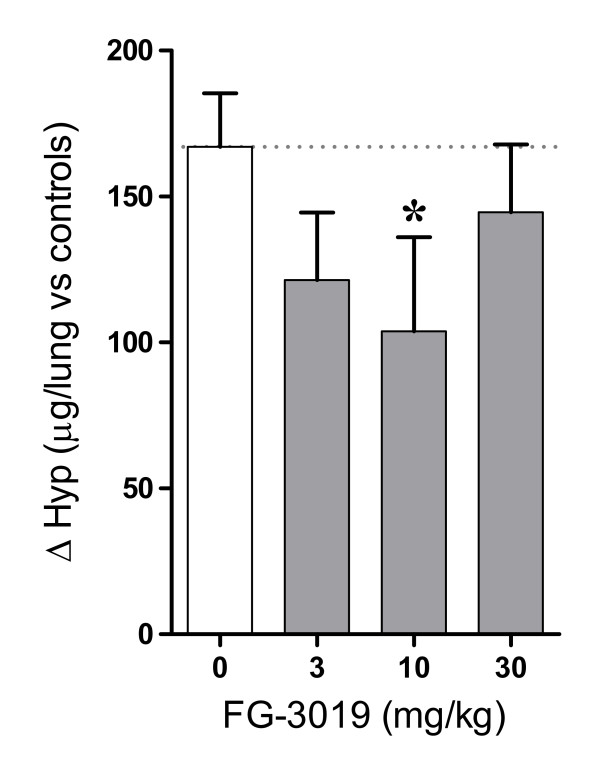
**FG-3019 inhibits collagen deposition in lungs of bleomycin-treated mice**. Pulmonary hydroxyproline (Hyp) content (μg/lung) of bleomycin-treated mice is expressed as the mean change from Hyp content of healthy control lungs (285 ± 8 μg/lung, mean ± SEM, *n *= 10). Mean Hyp content (μg/lung) increases for the 3, 10 and 30 mg/kg FG-3019 treatment groups (gray bars) were 27, 38 and 13% lower than for the bleomycin mice treated with vehicle (white bar), respectively. *P = 0.056 vs. bleomycin + vehicle (ANOVA, Fisher's LSD).

In control mice, bleomycin treatment increased mean ± SEM Hyp content to 452 ± 18 μg per lung (*n *= 6) compared with non-fibrotic saline-treated controls (285 ± 8 μg per lung; *n *= 10); a significant 59% increase of 167 μg per lung (*P *< 0.001). Compared with vehicle-treated bleomycin controls, FG-3019 anti-CTGF antibody treatment resulted in a consistent reduction in mean induced lung Hyp content; a 38% reduction in induced fibrosis in the 10 mg/kg FG-3019 treatment group exhibited a trend towards a treatment effect (*P *= 0.056) (Figure 5).

### FG-3019 Plasma Concentration

Circulating FG-3019 antibody levels were measured by ELISA to confirm FG-3019 exposure after intraperitoneal injection in the studies described above. In the bleomycin and UUO studies, mean ± SD FG-3019 plasma concentrations measured 48 hours after the final FG-3019 dose of 10 mg/kg were 184 ± 45.6 and 216 ± 71.8 μg/mL, respectively. Consistent with the lower daily and cumulative dose delivered in the neonatal TGF-β/CTGF synergy study, the FG-3019 plasma concentration 24 hours after the final 1.84 mg/kg dose was 21.8 ± 11.5 μg/mL.

## Discussion

Fibrosis, the excessive and persistent formation of scar tissue, is responsible for the morbidity and mortality associated with organ failure in a variety of chronic diseases affecting the lung, kidneys, eyes, heart, liver and skin. CTGF is strongly overexpressed in fibrotic tissue and is directly linked to the chronic fibrotic effects of TGF-β, VEGF, insulin-like growth factor, angiotensin II and other factors. CTGF acts as a cofactor with TGF-β to induce fibroblasts to become myofibroblasts that deposit collagen, ultimately resulting in organ scarring and dysfunction, and in the most severe forms, organ failure and death. Indeed, CTGF levels in tissue, blood or vitreal fluid have been shown to correlate with the degree and severity of fibrosis in many diseases [29]. Examples include diabetic nephropathy, glomerulosclerosis and IgA nephropathy (kidney), diabetic retinopathy and advanced macular degeneration (vitreal fluid), cirrhosis, biliary atresia and nonalcoholic steatohepatitis (serum, liver), congestive heart failure (myocardium), and various forms of pulmonary and skin fibrosis (serum, tissue) [5,29-32].

In the studies described here, the anti-CTGF antibody FG-3019 consistently diminished fibrotic responses in a novel model of CTGF and TGF-β synergy, and in previously described acute inflammatory models of lung and kidney fibrosis. Thus, the anti-fibrotic activity of FG-3019 in each of these models supports a key role for CTGF in mediating fibrogenesis. Indeed, FG-3019 alleviated the fibrosis associated with acute bleomycin and UUO injury even though the relatively severe nature of these acute injury models may limit the observable treatment benefit. It was not possible to determine from these models the specific nature of the anti-fibrotic effects of FG-3019, and additional studies will be needed to determine whether the FG-3019 action is exerted directly through effects on initiation, progression or resolution of fibrosis, or indirectly via effects on initial injury and/or early inflammatory response to injury [33]. Nevertheless, the UUO and bleomycin models were chosen because they have been extensively investigated, and because TGF-β and CTGF have each been implicated in their development and progression [16,22-24,28]. Thus, in the lung and kidney fibrosis models, endogenous TGF-β and CTGF were induced [28], and in the CTGF and TGF-β synergy model, recombinant exogenous TGF-β and CTGF were coadministered directly, with the latter approach resulting in profound encapsulating peritoneal sclerosis and systemic multiorgan fibrosis. Thus, each model provides a useful scenario for investigating CTGF/TGF-β cooperativity and the potential clinical utility of antifibrotic agents.

Previous findings indicated that coadministration of CTGF and TGF-β is required for the establishment of a persistent fibrotic effect in skin after subcutaneous administration [21,34-36]. In the current cooperative interaction model, intraperitoneal coadministration of TGF-β2 and CTGF resulted in the development of profound abdominal fibrosis and disseminated fibrosis in extraperitoneal organs, thus indicating induction of a systemic fibrotic response. Notably, induction of fibrosis in this model required concomitant exposure to both TGF-β and CTGF in a manner indicating functional synergy between these two cytokines in the fibrogenic response. These results indicate that CTGF is both necessary and sufficient to initiate fibrosis in the presence of increased TGF-β signaling, and that TGF-β is both necessary and sufficient to elicit fibrosis in the presence of increased CTGF. Because TGF-β is a potent inducer of endogenous CTGF, it is important to consider why the addition of further exogenous CTGF is required for TGF-β to elicit an overt fibrotic response. One possible explanation is that in the presence of pathologic fibrosis, TGF-β is indeed required, but fails to elicit enough endogenous CTGF on its own. This implies that independent pathways of CTGF regulation are also necessary. Moreover, it suggests that a CTGF threshold must be met to initiate or maintain fibrogenesis, and if so, then even partial inhibition of its activity to levels below a crucial threshold should provide anti-fibrotic benefit. Indeed, in the cooperative interaction model, FG-3019 anti-CTGF treatment reduced the overall level of fibrosis observed after CTGF and TGF-β coadministration in liver, kidney and lung. The magnitude of Hyp:Pro changes is influenced by pre-existing collagen content, the presence of less affected sample areas, and potential accumulation of non-collagenous proteins during fibrosis, whereas the histologic fibrosis scores measure the overall microscopic appearance of a tissue, including tissue areas that may be differentially affected, such as capsular or parenchymal regions. Thus, both measures support the qualitative conclusion that CTGF and TGF-β cooperate to promote tissue fibrosis, and that CTGF inhibition can suppress fibrogenesis.

Although the TGF-β/CTGF cooperative interaction model has properties suggestive of broad relevance to fibrotic disease, it may be specifically relevant to the formation of abdominal adhesions after surgery and to peritoneal fibrosis associated with dialysis procedures. Fibrosis in the TGF-β/CTGF cooperative interaction model was associated with profound peritoneal adhesions and signs of intestinal obstruction, a common presentation in patients diagnosed with postoperative peritoneal adhesions [37]. Moreover, CTGF mRNA is significantly increased in postoperative adhesions after peritoneal wounding in animal models [38] and in patients undergoing peritoneal dialysis [39]. Thus, it is likely that CTGF and TGF-β contribute to the formation of intra-abdominal adhesions in a cooperative and potentially targetable manner.

Together, these data provide both gain- and loss-of-function evidence that CTGF plays a crucial role in fibrotic tissue injury, because exogenous CTGF was required to initiate a fibrotic response in the presence of increased TGF-β levels, and because treatment with a CTGF-blocking antibody attenuated the fibrotic response in three independent models of pathologic fibrosis. Moreover, with regard to clinical utility, these data support the conclusion that the anti-CTGF monoclonal antibody, FG-3019, may be useful in controlling the progression of renal, pulmonary and hepatic fibrosis in patients with diseases such as diabetic nephropathy, systemi sclerosis or idiopathic pulmonary fibrosis. These results were obtained in studies of relatively limited treatment duration (14-20 days), as required by use of a human antibody in rodents, so that the added benefit of longer duration treatment regimens could not be assessed. In these studies, the optimum dose and regimen for FG-3019 administration was not fully established, and may differ by disease. However, the antifibrotic effects of FG-3019 have been reproduced in other studies, including comparisons where non-immune IgG groups were used [40].

CTGF exhibits a broad spectrum of biological activities, including effects on bone and cartilage development [41], cell adhesion and chemotaxis [42], and angiogenesis [43], none of which were addressed in these experiments. Moreover, fibrotic tissue remodeling also influences other clinically relevant processes, including cancer progression and organ-graft rejection. In addition to its antifibrotic effects, the FG-3019 anti-CTGF antibody used here has also been shown to attenuate tumor growth, metastasis and angiogenesis in mouse models of pancreatic cancer and to attenuate the anchorage-independent growth of CTGF-overexpressing pancreatic cancer cells [44-46]. Thus, taken together, these studies indicate that CTGF is a clinically relevant target in fibrosis and other disease states.

## Methods

### Animal selection and handling

Mice (Charles River Laboratories, Inc., Wilmington, MA, USA) were handled according to standards provided in the National Institutes of Health Guide and the Animal Welfare Act and to protocols approved by the Institutional Animal Care and Use Committee.

### UUO kidney fibrosis study

In total, 40 male Swiss Webster mice (32-35 g) were assigned to one of four groups (10 mice/group). On the first day of dosing, mice received an intraperitoneal dose of vehicle or FG-3019 (10 or 30 mg/kg), and 4 hours later, mice were anesthetized with isoflurane and UUO was performed on the left kidney [22]. The right (unoperated) kidney served as control. Thereafter, mice received vehicle or FG-3019 every other day to give a total of seven doses. Fourteen days after UUO, the surviving mice were anesthetized with isoflurane, blood was collected via the abdominal vein for plasma FG-3019 concentration by ELISA, and both the left and right kidneys were harvested. Mice that survived to the scheduled termination date were examined for successful ureteral ligation. Five mice with unsuccessful ligations (one from the UUO and vehicle group and four from the UUO and 10 mg/kg FG-3019 group) were excluded from analysis. Half of each kidney was processed for histopathologic analysis. The remaining half of each kidney was processed for determination of Hyp and Pro levels.

### Bleomycin-induced lung fibrosis study

In total, 40 male C57BL/6 mice (23-26 g) were assigned to one of four bleomycin-treated fibrosis groups (10 mice/group) and treated with FG-3019 (3, 10 or 30 mg/kg) or vehicle (control group). On the first dosing day, mice received an intraperitoneal dose of either vehicle or FG-3019, and 2 hours later received an intratracheal instillation of bleomycin (Bristol-Myers Squibb, Princeton, NJ, USA) at a dose of 0.08 U/50 μL/mouse under isoflurane anesthesia [24,47]. An additional non-fibrosis control group of 10 mice received an intratracheal instillation of saline, and was treated with vehicle. Thereafter, mice received vehicle or FG-3019 every other day to give a total of seven doses. During the dosing period, mice were observed daily, and weights were measured approximately three times per week. Animal weights in all bleomycin treatment groups were somewhat reduced versus saline non-bleomycin control, but final weights did not differ significantly between treatment groups. Similarly, bleomycin-associated mortality was similar between groups exposed to this agent. Fourteen days after the bleomycin instillation, the surviving mice were anesthetized with isoflurane. Blood was collected from the abdominal vein and placed in lithium-heparin tubes and spun at 4°C, then the plasma was frozen at -70°C for subsequent determination of FG-3019 concentration. All dissected lung lobes were quickly freed of non-parenchymal tissue, perfused with ice-cold saline, and hydrolyzed in 6 mol/l HCl at 105°C for 22 hours for subsequent whole-lung Hyp determination by high-performance liquid chromatography (HPLC) amino acid analysis. The standard measure of total lung Hyp in this model controls for the occasional inconsistent distribution of bleomycin injury associated with intratracheal instillation. Owing to the non-uniform nature of local bleomycin injury, histology samples were not collected.

### TGF-β/CTGF synergy study

Ten pregnant female Balb/c mice were used, with two dams assigned to each of five experimental groups to provide seven to 10 neonates per group. In each group, neonates were treated with up to two cytokines and with vehicle or FG-3019, as follows: no cytokine vehicle treatment control (healthy control; *n *= 10 neonates); TGF-β2 and vehicle (*n *= 7 neonates); CTGF and vehicle (*n *= 7 neonates); TGF-β2 and CTGF and vehicle (*n *= 10 neonates); and TGF-β2 and CTGF and FG-3019 (*n *= 8 neonates).

Mice aged 1 day old received their first intraperitoneal dose of FG-3019 (1.84 mg/kg) or vehicle (both in a volume of 0.05 mL), followed 1 hour later by intraperitoneal administration of recombinant human TGF-β2 (300 μg/kg) and/or CTGF (300 μg/kg) in a volume of 0.05 mL. This dosing regimen continued once daily for 20 consecutive days. Two mice died: one in the CTGF group (on day 10) and one in the group coadministered TGF-β2 and CTGF (on day 21, before scheduled termination). One day after the final FG-3019 dose, surviving mice were weighed and killed after blood collection for determination of plasma FG-3019 concentration. Organs were harvested for histopathologic analysis (kidney and liver) and determination of total Hyp and Pro (kidney, lung, liver and heart).

### Hyp and Pro determination

Hydroxyproline and proline were determined by the method of Palmerini *et al*. [48], except that L-azetidine 2-carboxylic acid (Sigma-Aldrich, St Louis, MO, USA) was substituted for 3,4-dehydroproline as an internal standard. Tissues were hydrolyzed in 6M HCl for 22 hours at 105°C. Samples underwent pre-column derivitization with *o*-phthalaldehyde and then 4-chloro-7-nitrobenzofuran (Sigma-Aldrich) to form fluorescent adducts of proline and hydroxyproline. The fluorescent adducts were separated and determined by reverse-phase HPLC followed by fluorometric detection.

### Histologic analysis

Tissue sections were independently examined in an unblinded manner by a licensed pathologist (Comparative Biosciences, Inc.). In the dual-cytokine synergy study, tissue sections were stained with hematoxylin and eosin and with trichrome, and a subjective but relative fibrosis severity score from 0 to 4 was assigned for each examined organ as given in Results, with small focal areas of trace fibrosis constituting 'minimal' fibrosis, and larger multifocal (that is,, non-diffuse) areas of fibroblasts and extracellular matrix accumulation constituting 'moderate' fibrosis.

### Test articles

FG-3019 was cloned, expressed and purified from a recombinant CHO cell line derived for this purpose. For bleomycin and UUO studies, FG-3019 was prepared in a vehicle solution consisting of 25 mmol/l L-histidine and 137 mmol/l sodium chloride, pH 6.0. For the TGF-β and CTGF synergy study, FG-3019 was prepared in phosphate-buffered saline (PBS; Cellgro™, Mediatech, Herndon, VA, USA). *E. coli*-derived TGF-β2 and baculovirus-derived recombinant human CTGF [49] were also prepared in PBS vehicle.

### Immunologic methods

Immunoblotting was carried out according to standard western blotting methods. FG-3019 or polyclonal antisera directed against either CYR61 or NOV were each used to determine the relative affinity of antibody preparations towards baculovirus-derived recombinant human CTGF, CYR61 and NOV. Rabbit polyclonal anti-NOV and anti-CYR61 antisera were generous gifts from Drs Maryvonne Laurent (Hôpital St. Antoine; Paris, France) and Ruth Lupu (UC Berkeley; Berkeley, CA, USA), respectively. For determinations of relative FG-3019 exposure, plasma FG-3019 antibody concentrations were determined by a sandwich ELISA using an exon-3 (VWC homology domain) CTGF peptide for capture of FG-3019 followed by detection with goat anti-human IgG linked to alkaline phosphatase.

### Statistical analysis

Statistical analyses were performed using the Sigma Stat program (SPSS Science, Chicago, IL, USA). Continuous data were analyzed by one-way analysis of variance with the Fisher's least significant difference test comparing all groups. Ordinal histologic fibrosis scores were analyzed by the non-parametric Kruskal-Wallis statistic with Dunn's multiple comparison post-hoc tests. The Fisher's exact test was used to analyze 2 × 2 contingency data for the presence or absence of histologic fibrosis. In the UUO study, Hyp:Pro ratios from uninjured contralateral control kidneys from all groups were pooled with the healthy control group. *P *< 0.05 was considered statistically significant, and unless otherwise indicated, within-group distributions are expressed as mean ± SEM.

## Competing interests

All authors are current employees of FibroGen, or were FibroGen employees when the studies were performed.

## Authors' contributions

Animal studies were designed and performed by QW, GG, BN and WZ, with input, materials and supporting analyses provided by WU, JG, MB, LX and TS. TS coordinated the preparation of the manuscript. NO, AL and DY supervised the studies. All authors read and approved the final manuscript.
